# Dr. Chatbot: Investigating the Quality and Quantity of Responses Generated by Three AI Chatbots to Prompts Regarding Carpal Tunnel Syndrome

**DOI:** 10.7759/cureus.81068

**Published:** 2025-03-24

**Authors:** Zachary J Buchman, Vincent R Savarino, Benjamin M Vinarski, Logan F Jay, David Phrathep, David Boesler

**Affiliations:** 1 Physical Medicine and Rehabilitation, Lake Erie College of Osteopathic Medicine, Bradenton, USA; 2 Internal Medicine, Lake Erie College of Osteopathic Medicine, Bradenton, USA; 3 Physical Medicine and Rehabilitation, University at Buffalo Jacobs School of Medicine and Biomedical Sciences, Buffalo, USA; 4 Physical Medicine and Rehabilitation, Brooks Rehabilitation Hospital, Jacksonville, USA; 5 Physical Medicine and Rehabilitation, Mayo Clinic Alix School of Medicine, Jacksonville, USA; 6 Osteopathic Medicine, Lake Erie College of Osteopathic Medicine, Bradenton, USA

**Keywords:** amboss, artificial intelligence in medicine, carpal tunnel syndome, chatgpt, compression neuropathy, google gemini, orthopedic sports medicine, peripheral entrapment neuropathy, physical medicine and rehabilitation, wrist injuries

## Abstract

Introduction

The objective of this study is to investigate the amount and accuracy of statements provided in answers by AI chatbots to prompts about carpal tunnel syndrome. To the authors’ knowledge, this is the first study to assess the answers provided by OpenAI™ ChatGPT-4o model, AMBOSS™ GPT, and Google™ Gemini to common patient-based questions regarding carpal tunnel, using UpToDate as a standard reference.

Objective

To determine which chatbot produces the most medically accurate responses. The authors hypothesize that the paid upgrade to Chat-GPT-4o (AMBOSS GPT) will have the most accurate responses compared to the two free chatbots, ChatGPT-4o and Google Gemini 1.5 Flash model.

Main outcome measures

The number of statements generated by each chatbot and the percentage of those statements that can be directly verified using exact quotations from supporting information available on UpToDate as of December 2024.

Results

There was a significant difference in terms of the number of average statements provided per prompt by the three chatbots, as GPT-4o produced 8.9 more statements compared to AMBOSS GPT (p = 0.0081916), GPT-4o produced 19.65 more statements compared to Gemini (p = 0.0000001), and AMBOSS GPT produced 10.75 more statements than Gemini (p = <0.0000001). There was also a significant difference in terms of the percentage of information provided by each chatbot that was able to be verified in AMBOSS GPT (85.97%) vs. GPT-4o (71.76%) and Gemini (73.53%), with differences of 14.22% (p = 0.0000002) and 12.44% (p = 0.0003969), respectively.

Conclusions

This study demonstrated that when looking at the three AI chatbots, AMBOSS GPT, GPT-4o, and Google Gemini, GPT-4o produced the most information per prompt; however, AMBOSS GPT provided a larger percentage of information that was able to be found supported within information available in UpToDate*.*

## Introduction

One of the greatest challenges in modern healthcare is the overwhelming amount of unvalidated medical information readily available on the internet [1]. This information forces healthcare workers to spend a substantial amount of time and energy explaining to patients that a benign or common problem may not be more severe than it appears [1]. With such a large amount of information available, it is difficult to discern which sources provide accurate, validated information. The introduction of AI chatbots into healthcare has an opportunity to rectify this issue and provide an opportunity to accurately educate patients as it relates to their health [2]. Since the early 2020s, AI chatbots have become a convenient means of producing succinct answers for individuals looking to gather information on any topic imaginable [3]. Although there is a great amount of information available through the use of AI, it is difficult to determine whether this information is any more factual than what is found through an online search engine, making it difficult to determine the role that AI could have in helping to relieve congested waiting rooms, month-long waits for appointments, and providing greater health equity for all [4].

Compared to the relatively untested AI chatbots, UpToDate is highly regarded as a gold standard medical reference that provides consensus knowledge developed using only the highest quality and most current clinical evidence in addition to their highly informed treatment algorithms [5,6]. This study aims to test the validity of statements provided by various AI chatbots on a well-known and highly researched disease, carpal tunnel syndrome (CTS), by comparing the statements made by AI chatbots to the medical reference, UpToDate. Currently, there are only two other studies by Amen et al. and Seth et al., which assess the ability of ChatGPT-4o to produce statements on the topic of CTS, but they fail to evaluate the accuracy of other AI chatbots and reference medical research studies directly rather than curated information from UpToDate [7,8]. Also, there is one other study that assesses the accuracy of chatbot statements in a similar style but not for CTS [9]. With these considerations, there is no other existing literature that has tested various AI chatbots (ChatGPT-4o, AMBOSS™ GPT, and Google™ Gemini) on the same prompts for CTS to make comparisons across widely known AI platforms that individuals may use to search for information. It ought to be acknowledged that there is an inherent subjectivity based on the semantic nature of data acquisition as well as minimal inferences made based on UpToDate quotations that did not directly provide support for these statements. However, measures were taken to maximize the objectivity of this study by using a large volume of statements recorded over the three AI chatbots and the usage of quotations directly from UpToDate to provide an accurate, highly regarded foundation of knowledge to support these statements. Lastly, given previous literature by Kuroiwa et al. in 2023 demonstrated flaws with generative AI’s ability to answer musculoskeletal questions with information able to be validated, this realm ought to be investigated using newer models [10]. By investigating the differences in the amount and quality of answers provided by ChatGPT-4o (created by OpenAI™ [11]), AMBOSS GPT (also created by OpenAI in collaboration with AMBOSS, designed to be a more clinically accurate version of GPT-4o [12]), and Google Gemini Flash model (the system that produces AI responses that rise to the top of a typical Google search [13]), the authors of this study hope to contribute to the development of a better understanding of the accuracy of medical information provided by chatbots to prompts given from a lay person's perspective.

## Materials and methods

On October 15, 2024, members of the research team posed 20 questions to ChatGPT-4o, AMBOSS GPT, and Google Gemini Flash model across four genres: general, diagnosis, treatment, and prognosis. The questions were asked verbatim across all chatbots and are displayed in Table 1.

**Table 1:** All questions posed to each chatbot All of the questions were posed to each chatbot and their associated genre. These questions were all posed in identical fashion to each chatbot, starting with “In as many words as you deem appropriate” and the following question.

Genre of prompt	“In as many words as you deem appropriate ____________”
General	What is carpal tunnel syndrome?
General	What causes carpal tunnel syndrome?
General	What are the symptoms of carpal tunnel syndrome?
General	What are some risk factors for carpal tunnel syndrome?
General	What can I do to prevent carpal tunnel syndrome?
Diagnosis	What tests can I do to tell if I have carpal tunnel syndrome?
Diagnosis	How do I know if I have carpal tunnel syndrome?
Diagnosis	I have numbness, tingling, and pain in my hand near my thumb. What is my diagnosis?
Diagnosis	The base of one of my thumbs is less thick than the other. What can cause this?
Diagnosis	My hand, near my thumb, tingles at night and when I type on the computer. What can cause this?
Treatment	What can I do to make carpal tunnel syndrome feel better?
Treatment	When should I see a doctor for carpal tunnel syndrome?
Treatment	What drugs help with carpal tunnel syndrome?
Treatment	What nondrug treatments help with carpal tunnel syndrome?
Treatment	When should I get surgery for carpal tunnel syndrome?
Prognosis	Can carpal tunnel syndrome go away on its own?
Prognosis	What are the long-term complications of untreated carpal tunnel syndrome?
Prognosis	Can carpal tunnel syndrome cause permanent damage?
Prognosis	Is treatment for carpal tunnel syndrome curative?
Prognosis	What are the side effects of drugs I can use to treat carpal tunnel syndrome?

Following posing these questions to each chatbot, the exact answers provided by the chatbots were copied and pasted into a spreadsheet. With the sum of answers provided by all chatbots in one central location, members of the research team began a process of extracting all the “statements presented as factual” (SPAFs), which are defined as components of the answers to a prompt that the chatbot presented as matter-of-fact statements. An example of statement extraction is displayed in Table 2 for the question “What is carpal tunnel syndrome?”. Also, the standard rules followed by the research team during this process for more nuanced SPAF extractions are displayed in Table 3.

**Table 2:** Example of SPAF extraction An example of SPAF extraction from a provided answer. Each SPAF is denoted using subscripts within the answer and correlates to the numbered list on the right. SPAF, statements presented as factual

Question: What is carpal tunnel syndrome?	Extracted SPAFs
Provided answer: Carpal tunnel syndrome is a condition that occurs when the median nerve, which runs through the carpal tunnel in your wrist_1_, is compressed_2_. This compression can cause numbness_3_, tingling_4_, pain_5_, and weakness_6_ in your hand.	1. The median nerve runs through the carpal tunnel in the wrist.
	2. Carpal tunnel syndrome is a condition that occurs when the median nerve is compressed.
		3. Compression of the median nerve can cause numbness in the hand.
	4. Compression of the median nerve can cause tingling in the hand.
		5. Compression of the median nerve can cause pain in the hand.
	6. Compression of the median nerve can cause weakness in the hand.	

**Table 3:** Standard rules for SPAF extraction The standard rules followed by the research team during this process for more nuanced SPAF extractions with associated examples and explanations for each. SPAF, statements presented as factual

Rule	Example answer	Explanation
For answers containing “and”, both components of the statement are considered separate SPAFs and treated as such during UpToDate referencing.	“The median AND ulnar nerves are involved in wrist flexion”	In this scenario, there are two SPAFs: (1) The median nerve is involved in wrist flexion and (2) The ulnar nerve is involved in wrist flexion.
For answers containing “or”, both components of the sentence are considered as one combined SPAF and only one component needs to be supported for the whole SPAF to be considered supported during UpToDate referencing.	“Symptoms of carpal tunnel syndrome include numbness OR tingling in the hand”	In this scenario, there is one SPAF and as long as either “numbness in the hand” or “tingling in the hand” is able to be supported by information in UpToDate, the SPAF is considered supported.
When answers to questions are provided in bullet/numbered format, each bullet/number is a SPAF and the following explanation is at least one SPAF.	“Here are some signs and symptoms that may indicate you have carpal tunnel syndrome: (1) Numbness or tingling: This often occurs in the thumb, index, middle, and ring fingers”	In this scenario, there are two SPAFs: (1) Numbness or tingling may indicate someone has carpal tunnel syndrome and (2) Numbness or tingling indicating carpal tunnel syndrome often occurs in the thumb, index, middle, and ring finger distribution.
When examples are provided in parentheses, these are separate SPAFs.	“Medications including NSAIDs (e.g., ibuprofen) have not been proven to be effective in relieving carpal tunnel syndrome pain”	In this scenario, there are two SPAFs: (1) NSAIDS have not proven to be effective in relieving carpal tunnel pain and (2) Ibuprofen is an NSAID.

After all SPAFs had been extracted from all provided answers, the next step was manual verification of each SPAF using all available content provided in UpToDate (Wolters Kluwer) as of December 2024, an online medical reference widely regarded as a gold standard online medical reference due to its highly rigorous, expert-led, editorial process. This reference was chosen as the sole reference for medical accuracy due to its paramount depth, broadness, accuracy, and relevance when it comes to medical information:

“To create and update the clinical topics in UpToDate, our editorial staff performs comprehensive reviews of the medical literature and considers the quality of the study, the hierarchy of evidence, and its clinical relevance. At the top of the evidence hierarchy are meta-analyses or randomized trials of high methodological quality, followed by randomized trials with methodological limitations, then observational studies and unsystematic clinical observations (expert consensus or opinions). Inferences are stronger when the evidence is summarized in systematic reviews that present all relevant data” [5].

Using numerous pages within UpToDate, each SPAF was designated as either “supported” or “unsupported/not supported” in accordance with the content of UpToDate. For each SPAF designated as “supported”, a direct quotation in support of this designation from UpToDate was documented. Documentation of quotations from UpToDate was also done for SPAFs designated as “Not supported/Unsupported” because of directly contradicting information within UpToDate. For SPAFs in which neither a quotation supporting nor a quotation refuting the claim was able to be obtained, no quotation was documented, but a “Not Supported” status remained for the SPAF. After this process was completed for all prompts, at least one other member of the research team reviewed every entry, bringing attention to any errors in SPAF extraction, judgment, and justification, and raising any concerns that were subsequently discussed until there was agreement among the members of the research team. This process resulted in the creation of a highly detailed 233-page document containing every answer provided to each question, the SPAFs associated with each answer, each SPAF’s supported status, as well as evidence justifying each support designation in quotations with appropriate author commentary. This document can, and will, be readily provided at any request to the corresponding author of this manuscript or can be accessed via this provided hyperlink (https://tinyurl.com/5hf8v3ca).

After support-status designations were made for each SPAF, data were able to be generated and analyzed regarding the quantity and verifiability of the answer given by each chatbot. Using the number of SPAFs generated by each chatbot for each prompt, a single-factor ANOVA analysis with post hoc t-tests was completed to determine if there were any significant differences between the chatbots in terms of the average number of SPAFs produced for each prompt, and if there were a difference, between which chatbots were there significant differences [14].

To conduct a similar analysis investigating the differences in verifiability among the chatbots, all SPAFs with a “supported” designation were labeled with a “1” and those “non-supported” with a “0”, creating a binary system of data points, with one data point for every SPAF produced by each chatbot. This allowed for another single-factor ANOVA analysis with post hoc t-tests to be conducted analyzing the differences among the chatbots in terms of their percentage of SPAFs able to be verified across all prompts [14].

Using this same binary data, the data were then segregated within the four genres of questions, providing data that were used to conduct four more single-factor ANOVA analyses with post hoc t-tests to investigate for differences among the chatbots in terms of their percentage of SPAFs able to be verified, but within specific genres of questions. In the results section, the genre-specific findings are presented, offering insight into how the three chatbots compared in terms of the average amount of information provided per prompt, the extent to which this information was supported by UpToDate, and whether differences existed based on the genre of the prompt [14].

## Results

**Figure 1:**
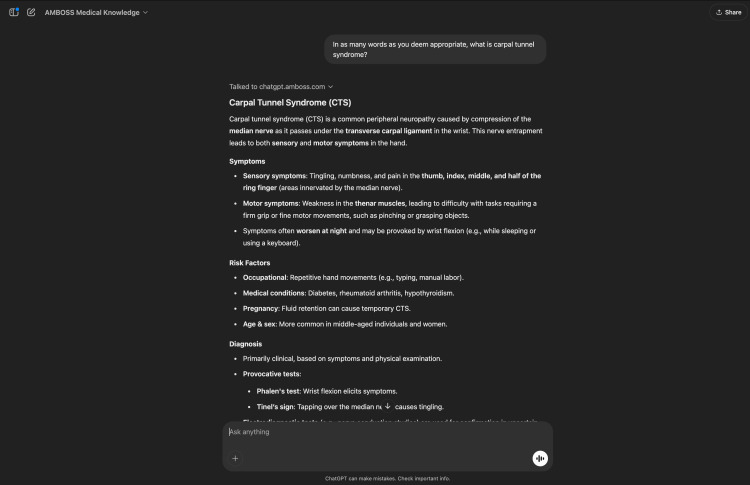
Screenshot illustrating the AMBOSS GPT interface and response to prompt #1

**Figure 2:**
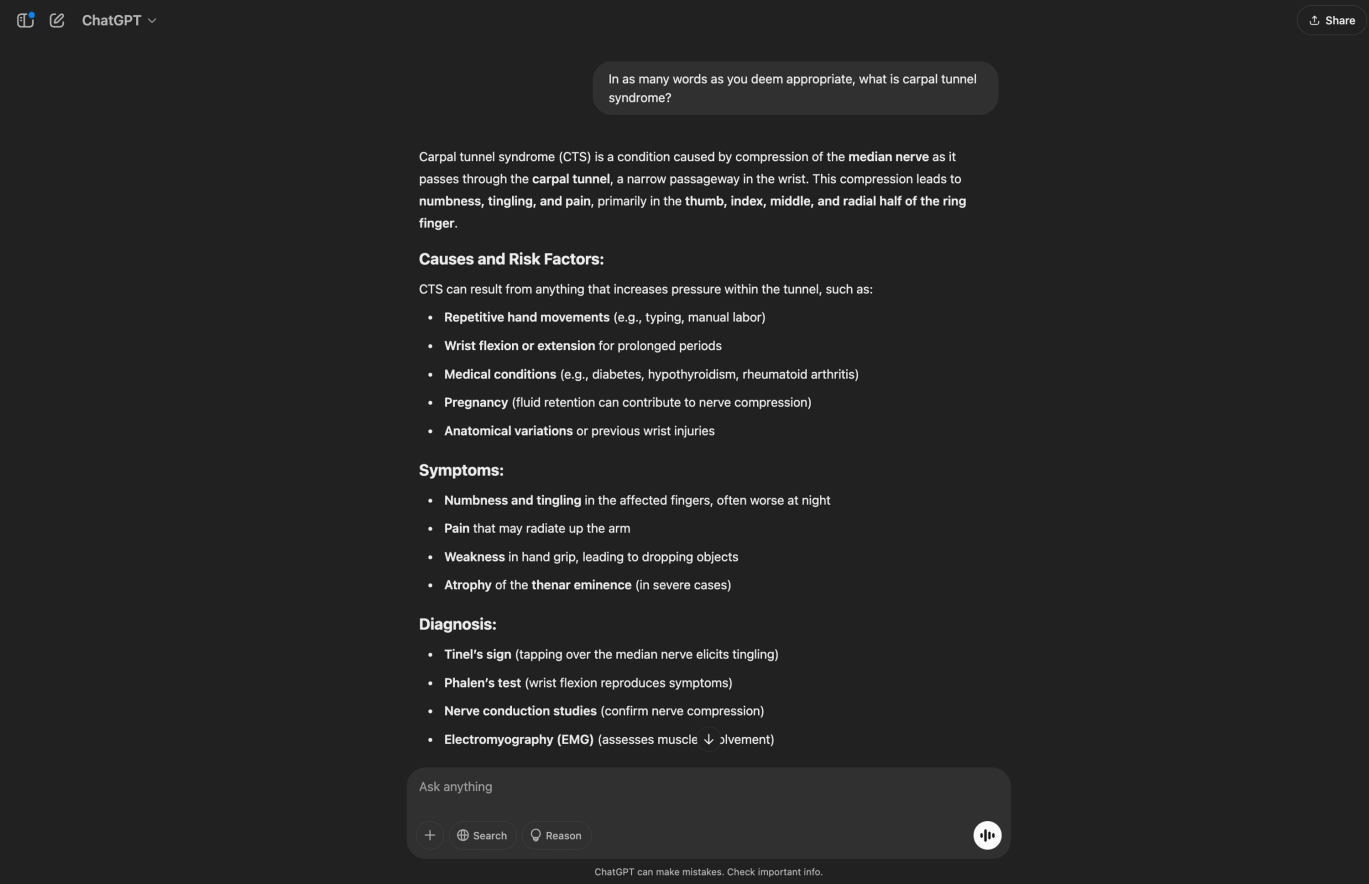
Screenshot illustrating the ChatGPT-4o interface and response to prompt #1

**Figure 3:**
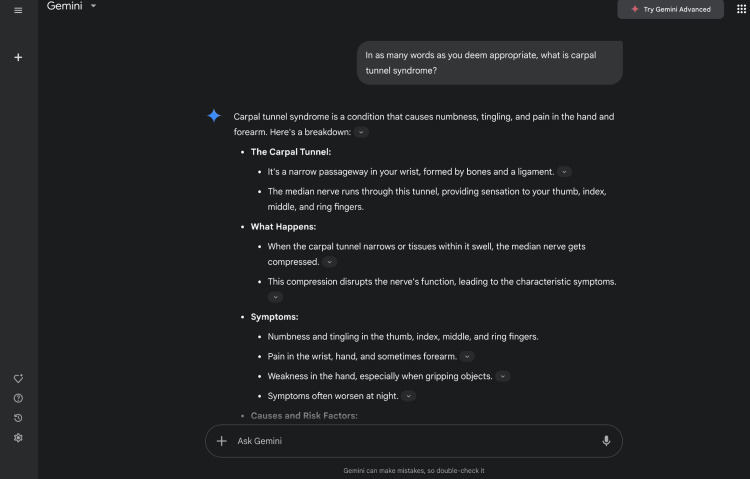
Screenshot illustrating the Google Gemini interface and response to prompt #1

To illustrate examples of the manner in which prompts were provided and answers were given, Figure 1, Figure 2, and Figure 3 display the interfaces of each chatbot to the same prompt with the names of each chatbot clearly visible.

A summary of acquired data is displayed in Table 4. With 1,118 SPAFs being able to be extracted from the responses of all three chatbots to all 20 prompts, a total of 860 (77%) of these SPAFs were able to be verified with supporting evidence in UpToDate. Data is also available in Table 4, displaying a breakdown of the quantity and verifiability of SPAFs in a prompt genre-specific manner.

**Table 4:** Summary of data A table displaying the raw data collected after SPAF extraction and verification processes, resulting in 1,118 SPAFs able to be extracted from the three chatbots’ responses to 20 prompts each. SPAF, statements presented as factual

Chatbot (genre)	# of SPAFs	# of SPAFs able to be verified	# of SPAFs unable to be verified
AMBOSS GPT (all genres of prompts combined)	385	331	54
AMBOSS GPT (general)	113	94	19
AMBOSS GPT (diagnosis)	102	89	13
AMBOSS GPT (treatment)	75	64	11
AMBOSS GPT (prognosis)	95	84	11
GPT-4o (all genres of prompts combined)	563	404	159
GPT-4o (general)	118	94	24
GPT-4o (diagnosis)	119	110	9
GPT-4o (treatment)	136	66	70
GPT-4o (prognosis)	190	133	57
Gemini (all genres of prompts combined)	170	125	45
Gemini (general)	43	28	15
Gemini (diagnosis)	52	45	7
Gemini (treatment)	37	24	13
Gemini (prognosis)	39	28	11
Total	1,118	860	258

For comparison of the number of SPAFs provided by each chatbot, Table 5 displays the results of a single factor ANOVA, and Table 6 displays post hoc two-sample, two-tail t-tests, comparing the average number of SPAFs produced by the three chatbots to each of the 20 prompts. The results of this ANOVA demonstrate a statistically significant difference in terms of the average number of SPAFs produced among the three chatbots (p = <0.0000001). Post hoc t-tests revealed that this difference was present between all chatbots, including AMBOSS GPT (average of 19.25 SPAFs per prompt) vs. GPT-4o (average of 28.15 SPAFs per prompt) (a difference of 8.9 SPAFs per prompt, p = 0.0081916); AMBOSS GPT (average of 19.25 SPAFs per prompt) vs. Gemini (average of 8.5 SPAFs per prompt) (a difference of 10.75 SPAFs per prompt, p = <0.0000001); and GPT-4o (average of 28.15 SPAFs per prompt) vs. Gemini (average of 8.5 SPAFs per prompt) (a difference of 19.65 SPAFs per prompt, p = 0.0000001). These findings demonstrate a significant difference among all three chatbots in terms of the average quantity of information provided by each chatbot to each prompt, with ChatGPT-4o producing the most, Google Gemini producing the least, and AMBOSS GPT falling in between.

**Table 5:** Results of single factor ANOVA comparing the average number of SPAFs produced per prompt among the three chatbots * Signifies a statistically significant difference (p < 0.05) SPAF, statements presented as factual

Chatbot	Average number of SPAFs per prompt	Variance	F	F _crit_	p-Value
AMBOSS GPT	19.25	9.4605263	27.2431195	3.1588427	<0.0000001*
GPT-4o	28.15	174.0289474			
Gemini	8.5	9.7368421			

**Table 6:** Post hoc two-sample t-test, two tails comparing the average number of SPAFs produced per prompt * Signifies a statistically significant difference (p < 0.05) SPAF, statements presented as factual

Between which groups	AMBOSS GPT vs. GPT-4o	AMBOSS GPT vs. Gemini	GPT-4o vs. Gemini
p-Value	0.0081916*	<0.0000001*	0.0000001*

Comparing the differences in verifiability of SPAFs provided by each chatbot, Table 7 displays the results of a single factor ANOVA, and Table 8 displays post hoc two-sample, two-tail t-tests, comparing the percentages of SPAFs produced by the three chatbots to each of the 20 prompts able to be found supported in UpToDate. The results of this ANOVA demonstrate a statistically significant difference in terms of the percentages of SPAFs produced by the three chatbots, able to be found supported (p = 0.0000010). Post hoc t-tests revealed that this difference was present between some, but not all, chatbots; specifically, a significant difference was found between AMBOSS GPT (85.97% of SPAFs found to be supported) vs. GPT-4o (71.76% of SPAFs found to be supported) (a difference of 14.22%, p = 0.0000002) and AMBOSS GPT (85.97% of SPAFs found to be supported) vs. Gemini (73.53% of SPAFs found to be supported) (a difference of 12.44%, p = 0.0003969), with GPT-4o vs. Gemini producing p > 0.05. These findings demonstrate a strong, significant difference between AMBOSS GPT (which had the greatest percentage of SPAFs able to be verified) and the other two chatbots but not between GPT-4o and Gemini, in terms of the percentage of information provided by each chatbot to each prompt able to be verified.

**Table 7:** Results of single factor ANOVA comparing the total percentage of SPAFs able to be verified (all genres combined) * Signifies a statistically significant difference (p < 0.05) SPAF, statements presented as factual

Chatbot	Percentage of SPAFs able to be verified	Variance	F	F _crit_	p-Value
AMBOSS GPT	85.97%	0.120901	13.9696389	3.0037955	0.0000010*
GPT-4o	71.76%	0.2030176			
Gemini	73.53%	0.1957884			

**Table 8:** Post hoc two-sample t-test, two tails comparing the total percentage of SPAFs able to be verified (all genres combined) * Signifies a statistically significant difference (p < 0.05) SPAF, statements presented as factual

Between which groups	AMBOSS GPT vs. GPT-4o	AMBOSS GPT vs. Gemini	GPT-4o vs. Gemini
p-Value	0.0000002*	0.0003969*	0.65213

To investigate differences among the chatbots in terms of the verifiability of the SPAFs they provided, with respect to the genre of prompt, the results of single factor ANOVAs with post hoc two-sample, two-tail t-tests are displayed, comparing the percentages of SPAFs found to be supported in UpToDate, produced by the three chatbots for each of the five prompts. The results of the ANOVA investigating “general” prompts, presented in Table 9, demonstrate a statistically significant difference in terms of the percentages of SPAFs produced by the three chatbots, able to be found supported (p = 0.0454533). Post hoc t-test results displayed in Table 10 reveal that this difference was present between one pair of chatbots; specifically, a significant difference was found between AMBOSS GPT (83.19% of SPAFs found to be supported) and Gemini (65.12% of SPAFs found to be supported) (a difference of 18.07%, p = 0.0144173), with no significant difference between AMBOSS GPT and GPT-4o (p > 0.05) and GPT-4o and Gemini (p > 0.05).

**Table 9:** Results of single factor ANOVA comparing the total percentage of SPAFs able to be verified (general prompts only) * Signifies a statistically significant difference (p < 0.05) SPAF, statements presented as factual

Chatbot	Percentage of SPAFs able to be verified	Variance	F	F _crit_	p-Value
AMBOSS GPT	83.19%	0.1411188	3.1265962	3.0290936	0.0454533*
GPT-4o	79.66%	0.1634072			
Gemini	65.12%	0.2325581			

**Table 10:** Post hoc two-sample t-test, two tails comparing the total percentage of SPAFs able to be verified (general prompts only) * Signifies a statistically significant difference (p < 0.05) SPAF, statements presented as factual

Between which groups	AMBOSS GPT vs.GPT-4o	AMBOSS GPT vs. Gemini	GPT-4o vs. Gemini
p-Value	0.4935626	0.0144173*	0.05720083

The results of the ANOVA investigating “diagnosis” prompts, presented in Table 11, demonstrated no statistically significant difference in terms of the percentages of SPAFs produced by the three chatbots, able to be found supported (p = 0.3528091). Post hoc t-tests displayed in Table 12 confirmed this with all tests between the three chatbots producing p-values >0.05.

**Table 11:** Results of single factor ANOVA comparing the total percentage of SPAFs able to be verified (diagnosis prompts only) * Signifies a statistically significant difference (p < 0.05) SPAF, statements presented as factual

Chatbot	Percentage of SPAFs able to be verified	Variance	F	F _crit_	p-Value
AMBOSS GPT	87.25%	0.1123083	1.0458585	3.0292181	0.3528091
GPT-4o	92.44%	0.0705028			
Gemini	86.54%	0.1187783			

**Table 12:** Post hoc two-sample t-test, two tails comparing the total percentage of SPAFs able to be verified (diagnosis prompts only) * Signifies a statistically significant difference (p < 0.05) SPAF, statements presented as factual

Between which groups	AMBOSS GPT vs. GPT-4o	AMBOSS GPT vs. Gemini	GPT-4o vs. Gemini
p-Value	0.2013056	0.9355937	0.2417910

The results of the ANOVA investigating “treatment” prompts, presented in Table 13, demonstrate a statistically significant difference in terms of the percentages of SPAFs produced by the three chatbots, able to be found supported (p = 0.0000004). Post hoc t-tests displayed in Table 14 revealed that this difference was present between some, but not all, chatbots; specifically, a significant difference was found between AMBOSS GPT (85.33% of SPAFs found to be supported) vs. GPT-4o (48.53% of SPAFs found to be supported) (a difference of 36.80%, p = <0.0000001) and AMBOSS GPT (85.33% of SPAFs found to be supported) vs. Gemini (64.86% of SPAFs found to be supported) (a difference of 20.47%, p = 0.0127737), with no significant difference between GPT-4o vs. Gemini (p > 0.05).

**Table 13:** Results of single factor ANOVA comparing the total percentage of SPAFs able to be verified (treatment prompts only) * Signifies a statistically significant difference (p < 0.05) SPAF, statements presented as factual

Chatbot	Percentage of SPAFs able to be verified	Variance	F	F _crit_	p-Value
AMBOSS GPT	85.33%	0.1268468	15.566938	3.032663	0.0000004*
GPT-4o	48.53%	0.251634			
Gemini	64.86%	0.2342342			

**Table 14:** Post hoc two-sample t-test, two tails comparing the total percentage of SPAFs able to be verified (treatment prompts only) * Signifies a statistically significant difference (p < 0.05) SPAF, statements presented as factual

Between which groups	AMBOSS GPT vs. GPT-4o	AMBOSS GPT vs. Gemini	GPT-4o vs. Gemini
p-Value	<0.0000001*	0.0127737*	0.0786423

The results of the ANOVA investigating “prognosis” prompts, presented in Table 15, demonstrated a statistically significant difference in terms of the percentages of SPAFs produced by the three chatbots, able to be found supported (p = 0.0023316). Post hoc t-tests displayed in Table 16 revealed that this difference was present between some, but not all, chatbots; specifically, a significant difference was found between AMBOSS GPT (88.42% of SPAFs found to be supported) vs. GPT-4o (70.00% of SPAFs found to be supported) (a difference of 18.42%, p = 0.00053874) and AMBOSS GPT (88.42% of SPAFs found to be supported) vs. Gemini (71.79% of SPAFs found to be supported) (a difference of 16.63%, p = 0.01813752), with no significant difference between GPT-4o vs. Gemini (p > 0.05).

**Table 15:** Results of single factor ANOVA comparing the total percentage of SPAFs able to be verified (prognosis prompts only) * Signifies a statistically significant difference (p < 0.05) SPAF, statements presented as factual

Chatbot	Percentage of SPAFs able to be verified	Variance	F	F _crit_	p-Value
AMBOSS GPT	88.42%	0.1034714	6.1771244	3.0238647	0.0023316*
GPT-4o	70.00%	0.211111			
Gemini	71.79%	0.2078273			

**Table 16:** Post hoc two-sample t-test, two tails comparing the total percentage of SPAFs able to be verified (prognosis prompts only) * Signifies a statistically significant difference (p < 0.05) SPAF, statements presented as factual

Between which groups	AMBOSS GPT vs. GPT-4o	AMBOSS GPT vs. Gemini	GPT-4o vs. Gemini
p-Value	0.00053874*	0.01813752*	0.82412258

Summary of significant findings

Combined, the three chatbots successfully had 860 out of 1,118 claims (77%) verified through direct quotes found in UpToDate (Table 4). Significant differences emerged in the quantity of SPAFs provided per prompt, with GPT-4o generating significantly more SPAFs per prompt than AMBOSS GPT, which, in turn, produced more than Gemini (Table 5, Table 6). Without considering the prompt genre, AMBOSS GPT had the highest percentage of verifiable SPAFs compared to the other two chatbots, while no significant difference was observed between GPT-4o and Gemini (Table 7, Table 8). When responding to general prompts about CTS, AMBOSS GPT provided a significantly higher percentage of verifiable SPAFs than Gemini (Table 9, Table 10). However, no differences were found among the chatbots regarding the percentage of verifiable SPAFs for prompts related to CTS diagnosis (Table 11, Table 12). For treatment-related prompts, AMBOSS GPT produced a significantly greater percentage of verifiable SPAFs compared to both Gemini and GPT-4o (Table 13, Table 14). Similarly, for prompts related to CTS prognosis, AMBOSS GPT demonstrated a significantly higher percentage of verifiable SPAFs than both Gemini and GPT-4o (Table 15, Table 16).

## Discussion

This study demonstrated that when considering AMBOSS GPT, GPT-4o, and Google Gemini, GPT4-o produced, on average, the most amount of information in response to prompts about CTS. However, AMBOSS GPT, the paid upgrade to GPT-4o designed to be more clinically accurate using AMBOSS as a medical reference, produced answers that were more frequently able to be found supported by information available in UpToDate, compared to the two other chatbots. This phenomenon of AMBOSS GPT’s superiority in terms of the verifiability of information produced was most pronounced for prompts regarding general topics of CTS when compared to Google Gemini as well as prompts about the treatment and prognosis of CTS when compared to both GPT-4o and Google Gemini. There were no differences seen among the three chatbots when it came to this metric for prompts about the diagnosis of CTS. What this means for individuals suffering from the symptoms of CTS is that while GPT-4o may be the option that produces the most amount of information when it comes to questions about CTS, the paid upgrade, AMBOSS GPT, produces information that is more likely to be medically accurate, and that Google Gemini may be the worst option of the three in terms of quantity and quality of information produced. Nevertheless, the findings of this study echo the other studies of a similar nature by Amen et al., Seth et al., and Johnson et al., where the chatbots were able to produce relatively comprehensive and accurate answers to medical questions from a patient’s perspective while also appropriately advocating for obtaining medical consultation when appropriate [7-9].

There were some common errors made by the chatbots that are worth mentioning. One of these would be that across all three chatbots, there was a common theme of mentioning the role of occupation and a person’s development of CTS has a well-defined relationship, which, according to UpToDate, is not the case: “The role of repetitive hand/wrist use and workplace factors in the development of CTS is controversial” [15]. Another similar repeated error on behalf of the chatbots was the repetition of NSAIDs being an appropriate treatment for CTS, which is also not the case according to UpToDate, “A 2003 systematic review found one randomized controlled trial that demonstrated no significant benefit for nonsteroidal anti-inflammatory drugs (NSAIDs) compared with placebo for improving CTS symptoms” [16]. These examples, along with only a handful of others, represented a large portion of the percentage of statements not able to be verified and, unfortunately, were repeated numerous times and imposed a disproportionate negative impact on the percentage of statements able to be verified.

In this study, there is a limit in terms of how fair it is to say that the percentage of statements able to be verified serves as an exact representation of how medically accurate the answers were. This is due to the use of only one source of gold standard reference, UpToDate, which provided a consistent measuring stick but left numerous times where the chatbots produced answers that may seem common sense to a practicing medical professional yet were unable to be verified. A good example of this was the definition of “thenar atrophy,” wherein although mentioned in UpToDate, a definition of this was never formally introduced, likely due to this being well understood among the intended audience of UpToDate. Instances like this reduced the overall percentage of statements able to be verified while not necessarily being invalid statements from a medical perspective. This makes a more appropriate interpretation of the statement mentioned in the results section, “a total of 76.92% of these SPAFs were able to be verified with supporting evidence in UpToDate,” be, “this study found that at an absolute minimum, 76.92% of the statements made by three chatbots were medically valid.” This interpretation considers that it is nearly certain that some statements given “unsupported” judgments were medically valid, while there is no reason to infer that any of the “supported” were invalid since they were backed up by supporting evidence from UpToDate. On the other hand, the use of more than one reference in this design could have introduced an increased risk for bias as, with any scientific topic, it is possible to come across conflicting information and leave it to UpToDate to decide which has the strongest evidence one way or another, limiting the possibility that a statement could be found supported while also unsupported later on, or vice versa. Another limitation of this study was the semantic nature of extracting SPAFs, which, although mitigated by objective rules to be followed, with over one thousand statements extracted, it would not be fair to say there is absolutely zero possibility for there to be differences in terms of how statements were extracted and interpreted. The rules mentioned in the discussion section, along with a system of double-checking each extraction, judgment, and verification, helped to mitigate this as much as possible, however.

One direction for future research would be to increase the number of chatbots investigated, as this study only used three. At the same time, at least two of the three are among the most popular used, as Google Gemini is the engine behind populating answers at the top of most Google searches just below the search bar/above links to relevant websites, and ChatGPT-4o has gained enormous popularity in recent years [3,13]. Unfortunately, AMBOSS GPT, the chatbot that produces the highest degree of verifiable information, is much less likely to be utilized by patients since it is a paid upgrade to GPT-4o and is designed for clinicians [12]. There is hope, however, as due to the design of AI chatbots, it is likely they will only improve due to their self-learning nature [17]. With that, it is likely that if another study of a similar nature were conducted decades from now, it would likely produce improved results [17].

## Conclusions

This study provides insight into the potential benefit of using AI chatbots for medical information but also highlights a significant emphasis on the requirement of caution in their use by the public for use in answering health-related questions. The integration of AI chatbots into clinical practices, or the use of them by the public for health information, therefore, requires a great deal of scrutiny before ultimately becoming commonplace. Before confidently endorsing these chatbots as a valid option for answering medical-style questions, several considerations for further studies are required. This could take place by validating information against a greater number of medical references than just UpToDate, as future research incorporating a more comprehensive list of sources would prove valuable. Also, a broader range of medical topics ought to be investigated beyond CTS to better generalize the accuracy of medical information overall provided by the chatbots. With the trajectory of AI’s role in modern society growing every day, it is becoming ever more important to investigate their products as their integration into important aspects of life, such as medicine, can have grave consequences if they are not understood to a maximal extent.
